# Polarization-Insensitive Surface Plasmon Polarization Electro-Absorption Modulator Based on Epsilon-Near-Zero Indium Tin Oxide

**DOI:** 10.1186/s11671-018-2446-0

**Published:** 2018-02-03

**Authors:** Lin Jin, Long Wen, Li Liang, Qin Chen, Yunfei Sun

**Affiliations:** 1 Key Lab of Nanodevices and Applications, Suzhou Institute of Nano-Tech and Nano-Bionics, Chinese Academy of Sciences(CAS), Suzhou, 215123 People’s Republic of China; 2 School of Electronic & Information Engineering, Suzhou University of Sciences and Technology, Suzhou, Jiangsu 215009 People’s Republic of China

**Keywords:** Surface plasmons, Plasmonics, Transparent conductor oxides, Epsilon-near-zero

## Abstract

CMOS-compatible plasmonic modulators operating at the telecom wavelength are significant for a variety of on-chip applications. Relying on the manipulation of the transverse magnetic (TM) mode excited on the metal-dielectric interface, most of the previous demonstrations are designed to response only for specific polarization state. In this case, it will lead to a high polarization dependent loss, when the polarization-sensitive modulator integrates to a fiber with random polarization state. Herein, we propose a plasmonic modulator utilizing a metal-oxide indium tin oxide (ITO) wrapped around the silicon waveguide and investigate its optical modulation ability for both the vertical and horizontal polarized guiding light by tuning electro-absorption of ITO with the field-induced carrier injection. The electrically biased modulator with electron accumulated at the ITO/oxide interface allows for epsilon-near-zero (ENZ) mode to be excited at the top or lateral portion of the interface depending on the polarization state of the guiding light. Because of the high localized feature of ENZ mode, efficient electro-absorption can be achieved under the “OFF” state of the device, thus leading to large extinction ratio (ER) for both polarizations in our proposed modulator. Further, the polarization-insensitive modulation is realized by properly tailoring the thickness of oxide in two different stacking directions and therefore matching the ER values for device operating at vertical and horizontal polarized modes. For the optimized geometry configuration, the difference between the ER values of two polarization modes, i.e., the ΔER, as small as 0.01 dB/μm is demonstrated and, simultaneously with coupling efficiency above 74%, is obtained for both polarizations at a wavelength of 1.55 μm. The proposed plasmonic-combined modulator has a potential application in guiding and processing of light from a fiber with a random polarization state.

## Background

Photonic integrated circuits (PICs) have made remarkable progress in the past few decades with the development of applications in the fields of optical communication, sensing, and imaging [1, 2]. Currently, considerable attention is being paid to downscale and reduce the power consumption of photonic devices to produce advanced PICs. Si photonics is deemed to be a promising solution for future high-speed on/off-chip optical interconnections. Typical Si waveguide modulators leverage electrically altering either the refractive or the absorptive properties of a material to modulate the transmission of light through a device. Due to the weak plasma dispersion effect of Si and the diffraction limit of the Si waveguides, the Si MZI modulators suffer from large footprints of ~ 10^3^–10^4^ μm^2^. The ring modulators with high Q resonance typically have more compact footprints of ~ 10^2^–10^3^ μm^2^ but lower optical bandwidth and tend to be more sensitive to temperature variation. Plasmonics provides an approach to miniaturize optical devices beyond the diffraction limit [3]. Alternatively, fully CMOS-compatible slot modulators or plasmonic modulators using Si as an active material are demonstrated recently [4, 5], and the high localization of a light field in the modulator can be achieved. However, the performance of the Si-based plasmonic modulator is still limited due to the small free carrier dispersion effect in Si layer (waveguide/structure).

Recently, transparent conductor oxides (TCOs), such as indium tin oxide (ITO), aluminum zinc oxide, and gallium zinc oxide, are emerging as attractive active materials for integrated electro-absorption (EA) modulators due to their electrically tunable permittivities [6–10]. Similar to Si-based field-effect MOS device where carrier accumulation is formed under an applied voltage bias, carrier density (*N*_ITO_) can be tuned at the ITO/dielectric interface with an applied bias. Obvious changes in refractive index of the ITO accumulation layer with a real part Δn = 0.092 and an imaginary part Δk = 0.27 have been experimentally reported at a free space wavelength of 1310 nm [10].When the real part of the permittivity of the ITO material is tuned to near zero, at a certain *N*_ITO_, which is referred as the “epsilon-near-zero” (ENZ) state, it has the maximal absorption loss due to the strong confinement of the guided mode [11]. In order to form the MOS capacitor structure and enhance the overlap between the optical field and the active material layer, slot waveguides [9, 12] and hybrid plasmonic waveguides [10] were adopted previously with the aim to strongly confine the guided mode in ITO and dielectric layer. Conventional plasmonic modulators including hybrid plasmonic modulators support only a transverse magnetic (TM) mode because the generation of the surface charge requires an electric field normal to the metal-dielectric interface and the slot waveguide with a strong optical field confinement support only a transverse electric (TE) mode in the slot region with low refractive index. For fiber optical communication applications, the light from a fiber usually has a random polarization state, and consequently, the signal-to-noise ratio will degrade when it couples into a polarization-sensitive optical modulator. The polarization-dependent loss could be very high in the case of plasmonic and slot ITO waveguide. Therefore, a polarization diversity system, such as polarization rotator [13–15], needs to be integrated into the circuit. However, it usually has a large coupling loss in the circuses. Accordingly, some ITO-based plasmonic modulators with low polarization dependent need to be considered. A compact EA modulator with a stack of TiN/HfO_2_/ITO/Cu deposited on a strip waveguide supports both TE and TM modes [11], but the difference between the extinction ratios of TE and TM reaches 0.9 dB/um, leading to 4% of the modulation efficiency. Therefore, a plasmonic modulator supporting both polarization modes with minimal ΔER is desired to realize the polarization-insensitive subwavelength light guiding and processing.

In this paper, the mode properties and the light modulation in a silicon waveguide cladded with Au/SiO_2_/ITO multilayers were investigated by numerical simulation. For both polarizations, highly concentrated plasmonic modes were supported in the Au/SiO_2_/ITO/Si stack either at the top or the sidewalls of the silicon core. The carrier dispersion effect in the ITO layer was used for modulation, which is tuned by the MOS capacitor structure formed by the stack. By tuning the carrier accumulation and mode field distribution in such a subwavelength waveguide, a modulation extinction ratio above 1.43 dB/μm can be achieved with a ΔER (a difference between the extinction ratios of two polarization modes) under 0.01 dB/μm. This result is promising to reduce the polarization-dependent loss in photonic integrated circuit.

## Methods

**Fig. 1:**
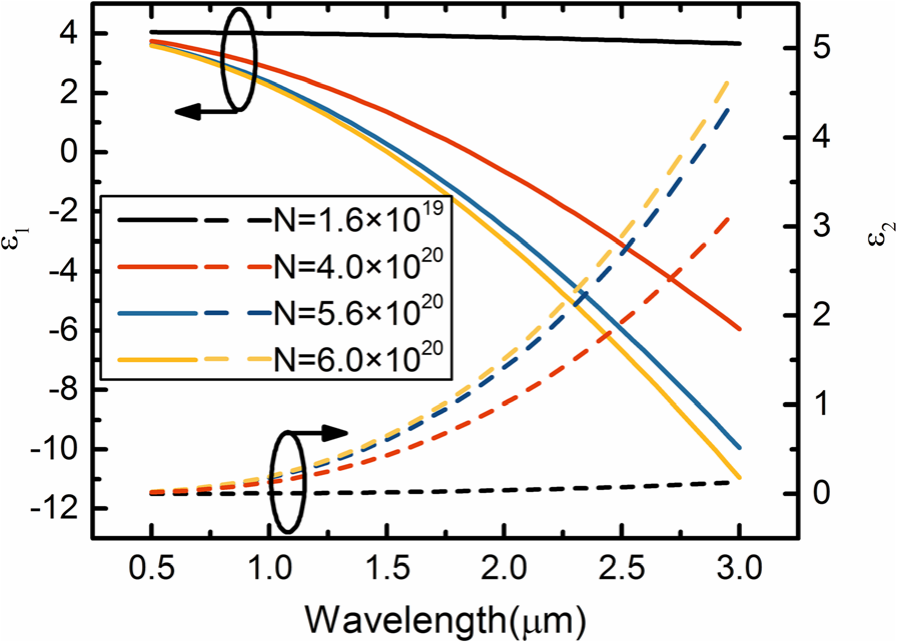
The calculated real part (*ε*_1_) and imaginary part (*ε*_2_) of the ITO’s permittivity as a function of the wavelength with different average electron concentration *N*_ITO_. The ENZ point of the wavelength is defined where *ε*_1_ crosses zero

In this paper, ITO is applied as an active material in the proposed modulator. The free carrier accumulation effect has been suggested as a promising approach for achieving high-speed plasmonic switching. In previous works, it has been confirmed that the refractive index of ITO can be altered significantly via charge carrier accumulation at ITO/dielectric interface in MOS capacitor structures [6, 16]. The permittivity of ITO can be treated by the Drude mode as

1 $$ \varepsilon ={\varepsilon}_{\infty }-\frac{N_{ITO}{e}^2}{\varepsilon_0{m}^{\ast }}\bullet \frac{1}{\omega^2+ i\omega \Gamma} $$

where *ε*_∞_ is the high-frequency permittivity, *Г* is the electron damping factor, *ω* is the angular frequency of light, *N*_ITO_ is the electron concentration of ITO material, m* is the effective mass, *e* is the electron charge, and *ε*_0_ is the permittivity of free space. It has been shown that the concentration of accumulated electrons maximizes at the ITO/dielectric interface and decrease quickly with the increasing distance from the interface [11]. Figure 1 plots the calculated real part (*ε*_1_) and imaginary part (*ε*_2_) of the ITO’s permittivity as a function of the wavelength at certain *N*_ITO_. One sees that, according to *N*_ITO_ = 6.0 × 10^20^ cm^− 3^, *ε*_1_ approaches zero at 1.55 μm. Physically, this represents a transition between a material exhibiting a dielectric response and a metallic response to incident light; this permittivity point is referred to as the ENZ point. ENZ materials lead to very large enhancement overlap in the optical field and the absorption layer. Meanwhile, the increase of carrier concentration also induces a corresponding increase of *ε*_2_, which increases the absorption loss in the carrier accumulation layer. In the later, we will compare the light modulation performance for various ITO EA modulators.

**Fig. 2:**
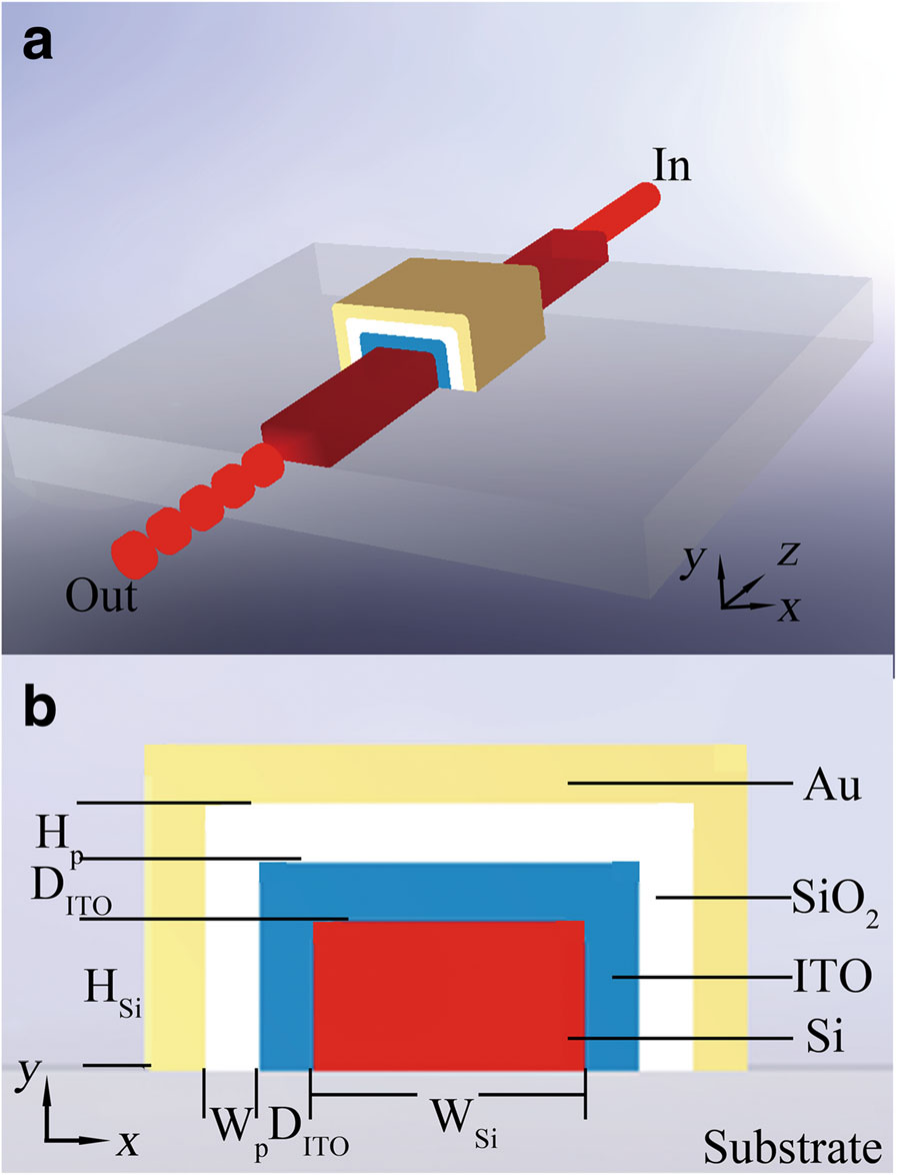
**a** 3D view and **b** cross-section of the proposed EA plasmonic modulator integrated with a stripe dielectric waveguide

To design a plasmonic modulator supporting and modulating both TE and TM guide modes, at least two metal-dielectric interfaces are required, one in the *x* direction and the other in the *y* direction. In this case, a plasmonic waveguide consisting of hybrid plasmonic waveguides in both vertical and horizontal directions is proposed. As shown in Fig. 2, the proposed modulator consists of a Si core with a width of *W*_Si_ and a height of *H*_Si_, a transparent conductive ITO layer with a thickness of *D*_ITO_, a SiO_2_ intermediate layer with a sidewall width of *W*_p_ and a height of *H*_p_, and a 100 nm thick (much thicker than the light penetration depth) Au cladding layer. Since the Si waveguide can be fabricated by e-beam lithographically and deep reactive-ion etching (DRIE), the thin ITO and SiO_2_ can be conformally deposited on the waveguide layer-by-layer using the well-developed pulsed laser deposition (PLD) method and PECVD method; the proposed modulator is CMOS backend-compatible. The HSPP wave is excited along the lower-refractive-index layer between the SiO_2_ and ITO layer, which can reduce the insertion loss effectively. Attributing to the quite different mode properties of these two types of plasmonic waveguides, the optical modulation is intrinsically different, but they could be designed to be polarization independent by optimizing the mode field distribution and the position of the active layer.

A finite-difference time-domain (FDTD) method is used to model the propagation properties numerically. A non-uniform mesh is used with a minimum spatial size of 0.2 nm. Perfectly matched layer (PML) boundaries are used to attenuate the field without the back reflection at all boundaries. The device has been designed to operate at a wavelength of 1.55 μm. The refractive indexes of silicon and silicon dioxide are 3.48 and 1.44, respectively, the dielectric constant of an Au cladding is assumed to be − 116.62 + 11.46i at 1.55 μm [17]. In this device, the metal/insulator/silicon (MIS) waveguide has excellent propagating properties, such as low loss and strong optical confinement in the waveguide beyond the limit of diffraction. Our previous work in full-cladding silicon plasmonic waveguides shows that this type of waveguide could support mode propagation of both polarizations and has very low difference of propagation constant [18].

## Results and discussion

**Fig. 3:**
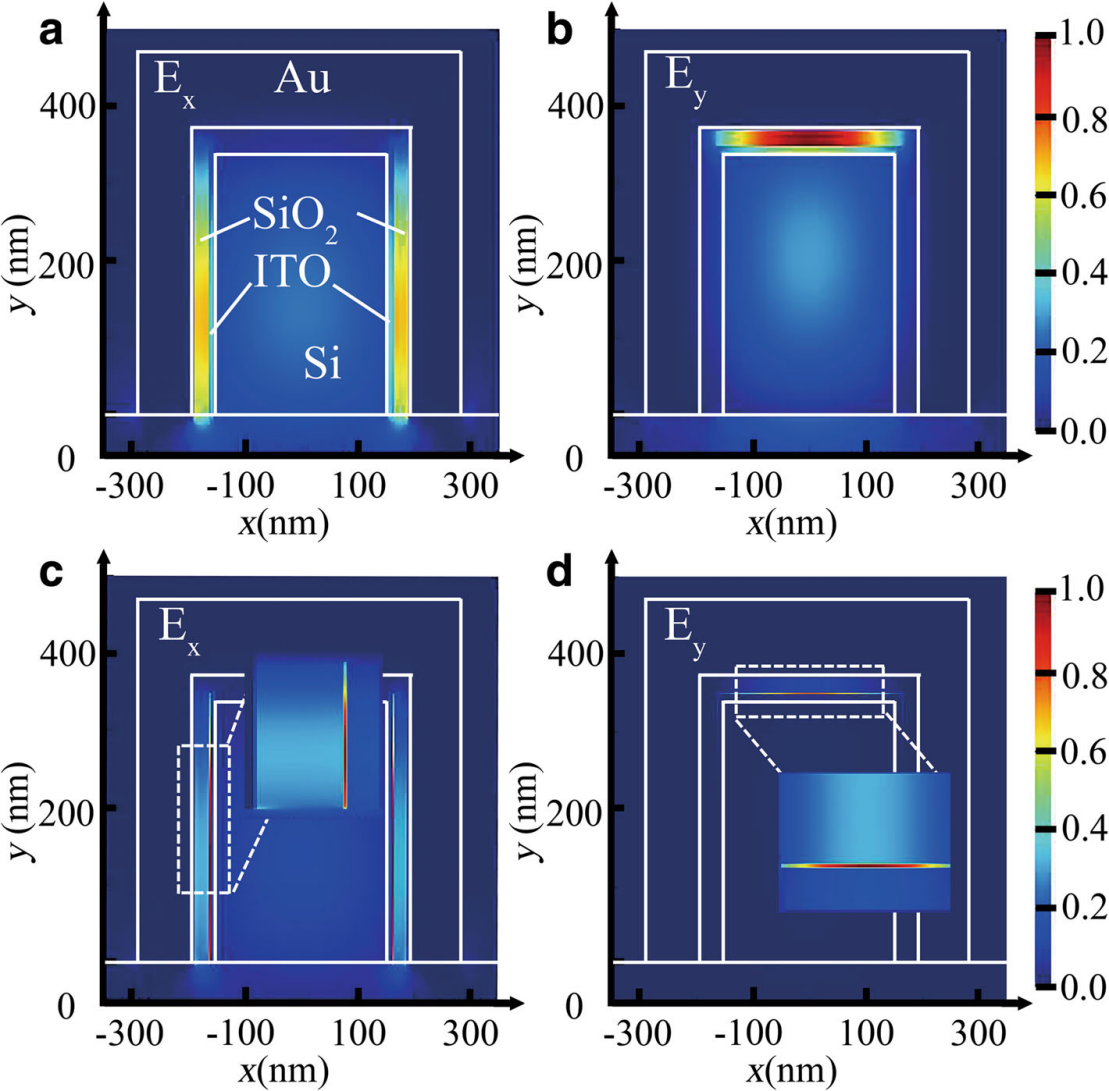
Electric field profiles *E*_x_ and *E*_y_ of the modulator for **a**–**b** “ON” state, *N*_ITO_ = 1.6 × 10^19^ cm^−3^, and **c**–**d** “OFF” state, *N*_ITO_ = 5.6× 10^20^ cm^−3^, respectively. **a** and **c** are for TE mode. **b** and **d** are for TM mode. The inserts show the zoomed in electric field density in ITO layer for the “OFF” state. *W*_Si_ = 310 nm, *H*_Si_ = 340 nm, *H*_p_ = 20 nm, *W*_p_ = 25 nm

To understand this variation in the hybrid plasmonic waveguide induced by NITO variation, which is defined as the average electron concentration in ITO layer, the electric field distributions *E*_x_ and *E*_y_ for an EA modulator are shown in Fig. 3. As shown in Fig. 3a, b, *N*_ITO_ = 1.6 × 10^19^ cm^−3^, *E*_x_ of the TE mode is confined at the two sidewalls of the SiO_2_ layer and *E*_y_ of the TM mode is confined at the top of the SiO_2_ layer, which offers a combination of both strong optical confinements significantly below the diffraction limit of light and relatively low light propagation loss [18, 19], defined as “ON” state. As shown in Fig. 3c, d, applying a voltage across the MOS capacitor structure, the carrier accumulation layers are induced at SiO_2_/ITO interfaces, *N*_ITO_ = 5.6 × 10^20^ cm^−3^. Due to the increasing of carrier density, the real part of the permittivity in both carrier accumulation layers decreases, which is lower than that in the SiO_2_ layers, the optical field will be pushed into the carrier accumulation layers. Meanwhile, due to the increase of the imaginary part of the permittivity in both the carrier accumulation layers as the *N*_ITO_ increases, the light propagation loss increases with the increase of the absorption loss in the carrier accumulation layers, which reaches the maximum at the ENZ point, i.e., “OFF” state.

**Fig. 4:**
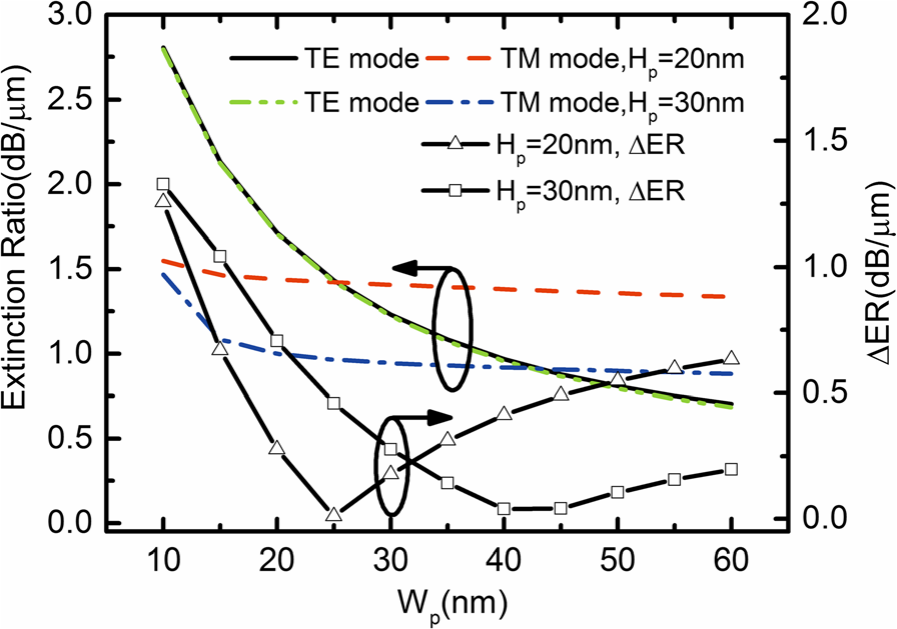
ER and ΔER of the EA modulator versus *W*_p_ at *H*_p_ = 20 and 30 nm

For a light modulator, ER and IL (insertion loss) are the two most important performance parameters. We define

2 $$ \mathrm{ER}=\frac{P_{\mathrm{out}}\left({V}_b={V}_{\mathrm{OFF}}\right)}{P_{\mathrm{out}}\left({V}_b={V}_{\mathrm{ON}}\right)} $$

3 $$ \mathrm{IL}=\frac{P_{\mathrm{in}}-{P}_{\mathrm{out}}\left({V}_b={V}_{\mathrm{ON}}\right)}{P_{\mathrm{in}}} $$

where *P*_out_ (*P*_in_) is the optical power at the output (input) of the device and *V*_b_ is the applied voltage at “ON” state (*V*_ON_) and “OFF” state(*V*_OFF_). In addition, the optical propagation loss (*α*) is defined as *α* = 4*πκ/λ*, *λ* is the operation wavelength and *κ* is the imaginary part of the complex effective index of the hybrid plasmonic mode. According to the calculation, *α* is mainly depending on the optical absorption in the carrier accumulation layers. The optical field in hybrid plasmonic waveguide is mostly confined in the low permittivity layer (SiO_2_ and ITO layer); therefore, the propagation loss would change with the varying of the SiO_2_ layer. To investigate the influence of the SiO_2_ layer dimensions on the modulation performance, ER and ΔER as a function of SiO_2_ layer have been discussed, as shown in Fig. 4. According to Fig. 4, ER of the TE mode gradually decreases with increasing *W*_p_ due to the overlap between the guided mode and the carrier accumulation layer decreased, leading to a small absorption in carrier accumulation layers. The ΔER reaches the minimum when *W*_p_ is slightly thicker than *H*_p_, due to a Si core with a rectangle cross-section and the optical absorption of two sidewalls.

**Fig. 5:**
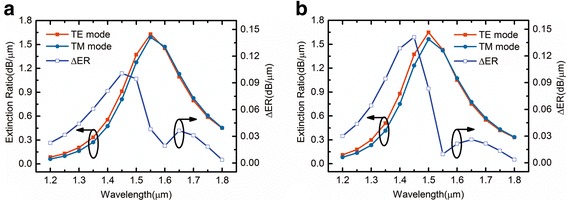
ER and ΔER as a function of wavelength for the EA modulator with **a** *N*_ITO_ = 5.6 × 10^20^ cm^−3^ and **b** *N*_ITO_ = 6.0 × 10^20^ cm^−3^

Figure 5 plots ER and ΔER as a function of wavelength for EA modulator with different *N*_ITO_. It can be seen that ERs and ΔER of EA modulator increase with wavelength increasing, reaching the maximum at a certain wavelength, and then ER decrease with wavelength further increasing, ΔER decreases and then reaches the minimum at a certain wavelength with wavelength further increasing. *N*_ITO_ for the maximum ER is near the ENZ point and *N*_ITO_ for the maximum ERs are at the ENZ point, for example, *N*_ITO_ = 6.0× 10^20^ cm^− 3^, the maximum ERs of both modes are 1.65 and 1.56 dB/μm at the wavelength of 1.50 μm, and the minimum ΔER is 0.009 dB/μm at the wavelength of 1.55 μm, which is our operation wavelength. For EA application, the condition when the maximum *α* is reached can be defined as the “OFF” state, and the condition when *α* is much smaller can be defined as the “ON” state. Moreover, for EA polarization-insensitive modulator, the condition when the minimum ΔER is reached should be paid much attention.

**Fig. 6:**
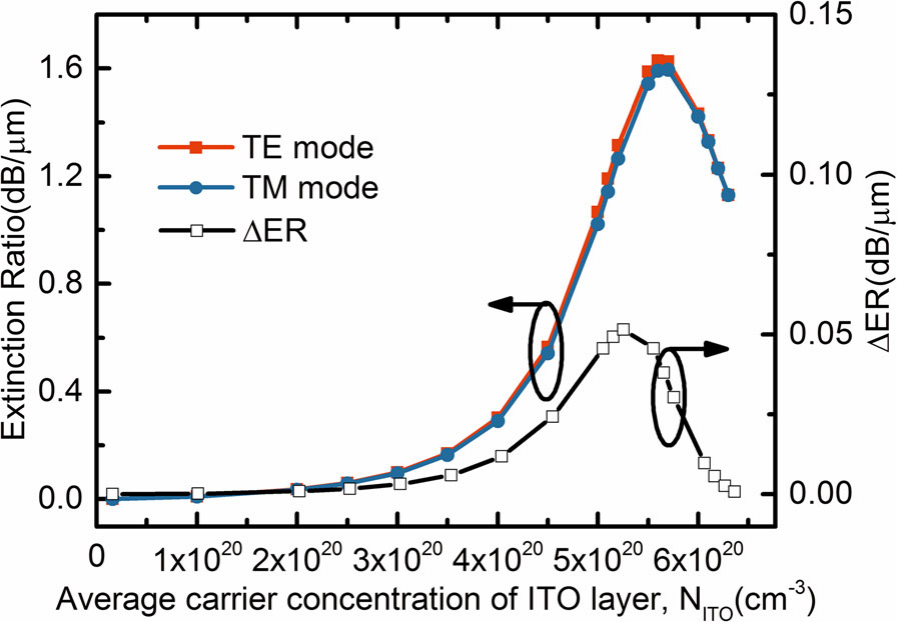
ERs and ΔER as a function of *N*_ITO_ for the EA modulator. *H*_Si_ = 340 nm, *W*_Si_ = 310 nm, *H*_p_ = 20 nm, *W*_p_ = 25 nm, *D*_ITO_ = 10 nm, *H*_Au_ = 100 nm

One sees that *N*_ITO_ in the carrier accumulation layer changes with the various applying voltage, resulting in the variation of the absorption and the electric field distribution. To understand the influences of the carrier accumulation layer for the EA modulation performance, ER and ΔER of the proposed modulator are calculated at operation wavelength. As seen in Fig. 6. ERs and ΔER of EA modulator increase with *N*_ITO_ increasing, reaching the maximum at a certain *N*_ITO_, and then decrease with *N*_ITO_ further increasing. The maximum ERs of TE and TM mode are 1.62 and 1.59 dB/μm, respectively. ΔER first increases with the increasing *N*_ITO_ and then decreases after reaching a maximum. One sees that, at ENZ point, ERs of both modes near the maximum, and ΔER is less than 0.01 dB/μm.

**Fig. 7:**
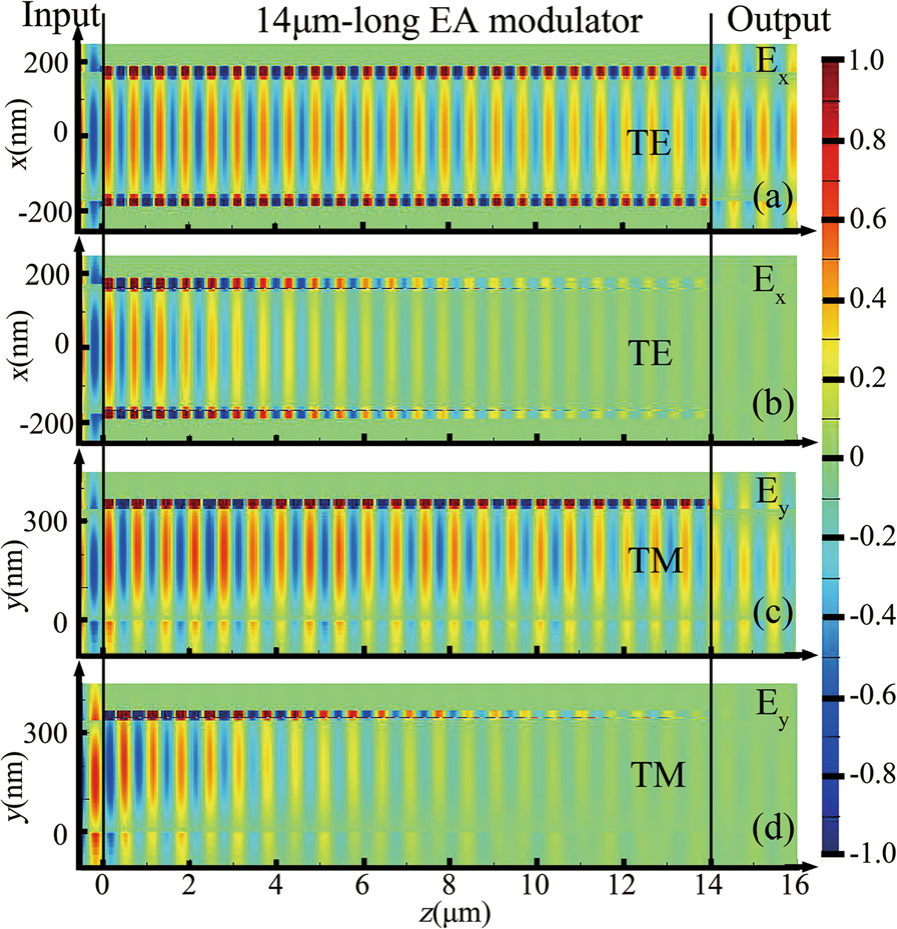
The field distributions of *E*_x_ for the TE mode **a**–**b** and *E*_y_ **c**–**d** for the TM mode along the *y*-cut and *x*-cut at the center of the Si waveguide. **a** and **c** are “ON” state. **b** and **d** are “OFF” state. *H*_Si_ = 340 nm, *W*_Si_ = 310 nm, *H*_p_ = 20 nm, *W*_p_ = 25 nm, *D*_ITO_ = 10 nm, *H*_Au_ = 100 nm

In order to demonstrate the device performance, 3D-FDTD simulations have been carried out for a 14-μm-long EA modulator. A 1.55-μm light with both TE and TM polarization is launched into the Si input waveguide, then propagating through the modulator, and finally coupled into the output Si waveguide. Figure 7a, b show the transverse electric field distributions along the *y*-cut at the center of the Si waveguide at “ON” state and “OFF” state. Figure 7c, d show the transverse magnetic field distributions along the *x*-cut at the center of the Si waveguide at “ON” state and “OFF” state. For “OFF” state, due to an excellent ΔER of 0.009 dB/μm, the lights at output of both TE and TM modes are balanced out with a 14-μm-long modulation length.

**Fig. 8:**
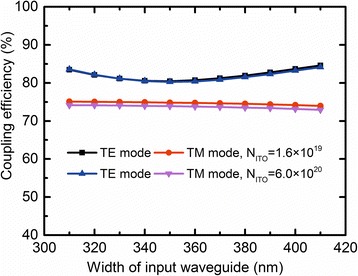
The CE between the plasmonic-combined waveguide and the Si waveguide as a function of the width for both TE and TM modes at “ON” state and “OFF” state. *H*_Si_ = 340 nm, *W*_Si_ = 310 nm, *H*_p_ = 20 nm, *W*_p_ = 25 nm, *D*_ITO_ = 10 nm, *H*_Au_ = 100 nm

For the design of HSPP modulator using in PICs, the Si waveguide width *W* (the height *H* = *H*_Si_ = 340 nm) has been optimized. By varying the waveguide width within the range where both TE and TM modes are supported, the coupling effective (CE) is calculated. From Fig. 7, some reflected light at the coupling interface is observed due to the mode mismatch in these two waveguides, resulting in a coupling loss. The mode mismatch between the Si stripe waveguide with a larger *n*_eff_ and the plasmonic-combined waveguide becomes large, resulting in the decrease of the coupling efficiency. Figure 8 shows the CE (defined as the radio of the power flux recorded in a plane behind the interface of two waveguides to the source) between the plasmonic-combined waveguide (*H*_p_ = 20 nm and *W*_p_ = 25 nm) and the Si waveguide as a function of width for both TE and TM modes. It can be seen that when *W* increases, the ΔCE (a difference between the coupling efficiency of two polarization modes) decreases, reaching its minimum at a certain width of the input Si waveguide, and then increases with rising tide of width of the input Si waveguide. As a consequence, the minimum ΔCE are 5.63% (“ON” state) and 6.38% (“OFF” state); therefore, the coupling efficiency is nearly polarization-insensitive with 80.46% for TE mode and 74.83% for TM mode at “ON” state.

## Conclusions

In summary, we presented an EA polarization-insensitive plasmonic waveguide modulator. The waveguide structure consists of hybrid waveguides in both *x* and *y* directions, where dual polarization modes are existing. The hybrid plasmonic waveguide forms a MOS capacitor where the carrier accumulations occur at dielectric-ITO interfaces when the doped-Si electrode is biased at a lower voltage than metal electrode. The light modulation is investigated by tuning the carrier density. A minimum ΔER of 0.009 dB/μm at the wavelength of 1.55 μm is demonstrated by simulation. This ΔER is lowest on record as we know. Furthermore, coupling efficiencies above 74% for both polarizations are obtained using a feeding silicon waveguide. These ITO EA plasmonic waveguide modulators could be an important building block for ultra-compact photonic integration. In future works, optimization of the geometry of the asymmetric coating with larger tolerance should be considered for the sake of ease of fabrication.
